# Metabolic Reprogramming by Hexosamine Biosynthetic and Golgi N-Glycan Branching Pathways

**DOI:** 10.1038/srep23043

**Published:** 2016-03-14

**Authors:** Michael C. Ryczko, Judy Pawling, Rui Chen, Anas M. Abdel Rahman, Kevin Yau, Julia K. Copeland, Cunjie Zhang, Anu Surendra, David S. Guttman, Daniel Figeys, James W. Dennis

**Affiliations:** 1 Lunenfeld-Tanenbaum Research Institute, Mount Sinai Hospital, 600 University Ave., Toronto ON M5G 1X5, Canada; 2 Department of Molecular Genetics, University of Toronto, Toronto ON M5S 1A8, Canada; 3 Ottawa Institute of Systems Biology, Department of Biochemistry, Microbiology and Immunology, Faculty of Medicine, University of Ottawa, Ottawa ON K1H 8M5, Canada; 4 CAS Key Lab of Separation Sciences for Analytical Chemistry, National Chromatographic Research and Analysis Center, Dalian Institute of Chemical Physics, Chinese Academy of Sciences, Dalian 116023, China; 5 Department of Genetics, Research Center, King Faisal Specialist Hospital and Research Center, Riyadh 11211, Kingdom of Saudi Arabia; 6 Centre for the Analysis of Genome Evolution & Function, University of Toronto, Toronto ON M5S 3B2, Canada; 7 Department of Chemistry, Faculty of Science, University of Ottawa, Ottawa ON K1N 6N5, Canada; 8 Department of Laboratory Medicine and Pathobiology, University of Toronto, Toronto ON M5S 1A8, Canada

## Abstract

*De novo* uridine-diphosphate-N-acetylglucosamine (UDP-GlcNAc) biosynthesis requires glucose, glutamine, acetyl-CoA and uridine, however GlcNAc salvaged from glycoconjugate turnover and dietary sources also makes a significant contribution to the intracellular pool. Herein we ask whether dietary GlcNAc regulates nutrient transport and intermediate metabolism in C57BL/6 mice by increasing UDP-GlcNAc and in turn Golgi N-glycan branching. GlcNAc added to the drinking water showed a dose-dependent increase in growth of young mice, while in mature adult mice fat and body-weight increased without affecting calorie-intake, activity, energy expenditure, or the microbiome. Oral GlcNAc increased hepatic UDP-GlcNAc and N-glycan branching on hepatic glycoproteins. Glucose homeostasis, hepatic glycogen, lipid metabolism and response to fasting were altered with GlcNAc treatment. In cultured cells GlcNAc enhanced uptake of glucose, glutamine and fatty-acids, and enhanced lipid synthesis, while inhibition of Golgi N-glycan branching blocked GlcNAc-dependent lipid accumulation. The N-acetylglucosaminyltransferase enzymes of the N-glycan branching pathway (Mgat1,2,4,5) display multistep ultrasensitivity to UDP-GlcNAc, as well as branching-dependent compensation. Indeed, oral GlcNAc rescued fat accumulation in lean Mgat5^−/−^ mice and in cultured Mgat5^−/−^ hepatocytes, consistent with N-glycan branching compensation. Our results suggest GlcNAc reprograms cellular metabolism by enhancing nutrient uptake and lipid storage through the UDP-GlcNAc supply to N-glycan branching pathway.

N-acetylglucosamine (GlcNAc) is found in the N-glycans that modify glycoproteins produced in the secretory pathway and in other glycoconjugates, many with ancient origins such as the GlcNAc polymer chitin found in arthropods, molluscs, insects and fungi^1^. *De novo* UDP-GlcNAc biosynthesis by the hexosamine biosynthetic pathway (HBP) requires glucose (Glc), glutamine (Gln), acetyl-coenzyme A (Ac-CoA) and uridine triphosphate (UTP), metabolites that are central to carbon, nitrogen, fatty-acid, and energy metabolism. In cell-culture, glucose depletion reduces UDP-GlcNAc levels, whereas excess glutamine increases UDP-GlcNAc^2^. A rate limiting step in HBP is the conversion of fructose-6-phosphate (Fru-6P) and Gln to glucosamine-6P (GlcN-6P) and glutamate by glutamine:fructose-6P-aminotransferase (Gfpt)^3^. Transgenic mice overexpressing Gfpt in the liver displayed obesity, enhanced glycogen storage, impaired glucose tolerance, and insulin resistance at 8 months of age^4^.

UDP-GlcNAc is a required substrate in multiple protein glycosylation pathways, thereby impacting the proteome widely. One the most pervasive is O-GlcNAcylation of cytoplasmic, nuclear and mitochondrial proteins, a dynamic modification associated with signaling and gene transcription^5^. Transgenic mice overexpressing O-GlcNAc transferase (OGT) in muscle and fat tissues display insulin resistance^6^. Transgene overexpression of Gfpt to elevate UDP-GlcNAc is reported to have a similar phenotype^7^, although the relationship between UDP-GlcNAc and metabolism appears to be more complex. For example, increasing O-GlcNAcylation globally with a selective inhibitor of O-GlcNAcase does not affect body-weight, induce insulin resistance, nor perturb glucose homeostasis in rodents or 3T3-L1 adipocytes^8^,^9^. Haploinsufficient mice for O-GlcNAcase (Oga^+/−^) display improved glucose tolerance, a lean phenotype, and resistance to high-fat diet-induced obesity^10^; opposite to the expectation of higher levels of protein O-GlcNAcylation. It is likely that the activity of multiple glycosylation pathways interact through a shared pool of UDP-GlcNAc in a complex and cooperative manner. In support of this idea, disruption of O-GlcNAc cycling in *C. elegans* perturbs nucleotide sugar pools and complex N-glycans^11^. Mutations in genes encoding Golgi N-glycan branching enzymes Mgat4a and Mgat5 disrupt glucose homeostasis in mice^12^,^13^. The N-acetylglucosaminyltransferases encoded by Mgat1, Mgat2, Mgat4a/b/c and Mgat5 each catalyze the transfer of GlcNAc from UDP-GlcNAc in a specific β-linkage to the trimannosyl core of glycoprotein N-glycans^14^. The N-glycan branching pathway is multistep ultrasensitive to UDP-GlcNAc due to a ~300-fold decline in affinity for this common donor substrate, moving down the pathway from Mgat1 to Mgat5^15^. With a *K*_*m*_ value of ~10 mM for UDP-GlcNAc, Mgat5 and the synthesis of tri- and tetra-antennary N-glycans is most sensitive to UDP-GlcNAc levels. Mgat5^−/−^ mice display adult phenotypes that may be linked in part through metabolism, including delayed oncogene-induced tumor progression^16^, autoimmune sensitivity^17^, depression-like behaviour^18^, lean body composition, resistance to weight-gain on high-fat diet, hypoglycemia, sensitivity to fasting, loss of adult stem cells and early aging^19^.

The GlcNAc branches are extended with galactose, fucose and sialic acid, generating N-glycan structures with affinity for galectins, C-type lectins and siglecs at the cell surface^20^. Galectins bind N-acetyllactosamine (Galβ1-4GlcNAcβ) branches on N-glycans, and their affinities for membrane glycoproteins are proportional to both extent of branching and N-glycan number, specified by NXS/T consensus site and encoded in protein sequence^15^. N-glycan branches mediate lectin binding in a cooperative manner to regulate glycoprotein dynamics and cell surface residency of cytokine receptors, as well as nutrient transporters^13^,^15^. For example, glucose transporter Glut4 has a single N-glycan and is retained at the cell surface in a UDP-GlcNAc-dependent manner^15^. Similarly, galectin-9 binds to the single multi-antennary N-glycan branch on pancreatic Glut2 and prevents its loss to endocytosis, thus allowing glucose import and glucose-stimulated insulin secretion^12^,^21^. Mgat4a^−/−^ mice fail to retain Glut2 on the surface of pancreatic β-cells, resulting in reduced glucose sensing and insulin secretion, and development of hyperglycemia^12^. Inducible overexpression of Mgat5 in Hek293 cells increased Gln uptake, intracellular metabolites, and cell growth in low glucose and glutamine medium^22^. Similarly, treatment of glucose-starved immortalized hematopoietic cells with GlcNAc promoted cell surface expression of IL-3 receptor and Akt and mTOR growth signaling, along with glutamine uptake, catabolism and support of lipid biosynthesis in a manner dependent on Golgi N-glycan branching^23^.

GlcNAc is commercially available as a dietary supplement, and oral GlcNAc in rats has shown no overt toxicity^24^,^25^. A study in children with treatment-resistant severe inflammatory bowel disease showed clinical improvement in a majority of cases following adjunct treatment with oral GlcNAc at 3–6 g/day (60–120 mg/kg/day)^26^. Oral GlcNAc at 0.25 mg/ml in drinking water, estimated at ~40 mg/kg/day, suppressed spontaneous autoimmune diabetes in non-obese diabetic mice when initiated prior to disease onset^27^. Importantly, oral GlcNAc increased UDP-GlcNAc supply and N-glycan branching on T cell glycoproteins *in vivo*^27^. Here we asked whether GlcNAc supplementation in drinking water of mice can be salvaged by HBP and interact through N-glycan branching to increase anabolic metabolism and weight-gain. We report that oral GlcNAc was taken up into the circulation and increased hepatic UDP-GlcNAc levels, N-glycan branching on hepatic glycoproteins, and weight-gain without affecting food intake, physical activity or energy expenditure. Furthermore, oral GlcNAc decreased the respiratory exchange ratio (RER), suggesting increased lipid catabolism concomitant with enhanced lipid accumulation. GlcNAc supplementation rescued lipogenesis and fat accumulation in Mgat5^−/−^ mice and primary hepatocytes. In cultured cells, GlcNAc enhanced uptake of glucose and glutamine, and promoted lipid accumulation in an N-glycan-dependent manner. Our results suggest that UDP-GlcNAc supply to Golgi N-glycan branching regulates the rates of nutrient uptake and lipid storage in adult mice.

## Results

### Oral GlcNAc Supplementation Alters Liver Metabolism

**Figure 1:**
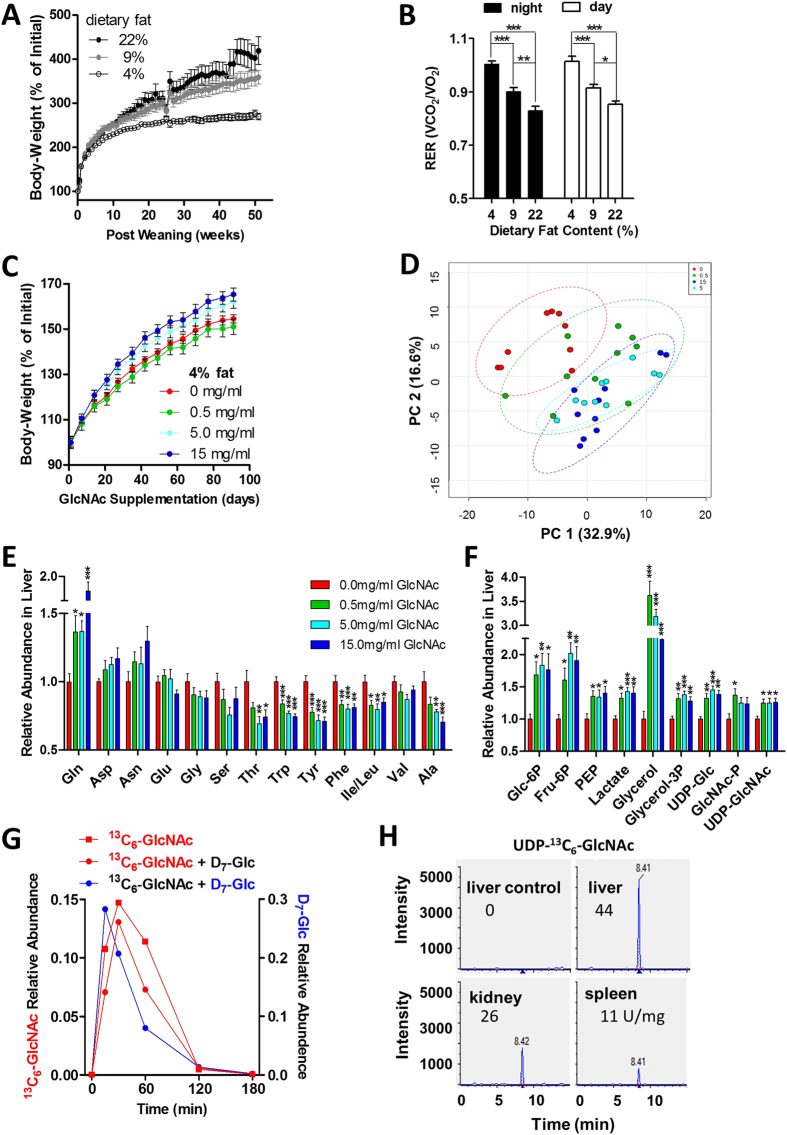
Oral GlcNAc increases tissue UDP-GlcNAc and promotes weight-gain in young mice. **(A)** Change in body-weight for wild-type C57BL/6 male mice on diets containing different percentages of fat. Data shown are mean ± SEM, n = 8–11. **(B)** Respiratory exchange ratio (RER) for night and day in mice fed different percentage fat diets for 50 weeks. Data shown are mean ± SEM, n = 8–11, analyzed by one-way ANOVA followed by Tukey’s multiple comparison test, with significant differences indicated as *p < 0.05, **p < 0.01 and ***p < 0.001. **(C)** Change in body-weight of C57BL/6 male mice on 4% fat diet beginning at 9 weeks of age with GlcNAc supplemented drinking water at 0.5, 5.0 and 15 mg/ml, n = 10, *p* < 0.001 ANOVA. **(D)** Relative abundance of ~150 liver metabolites measured by targeted LC-MS/MS and represented as principle component analysis at 90 days of GlcNAc treatment. Specific data for **(E)** amino acids and **(F)** glycolytic and gluconeogenic hepatic metabolites expressed as fold change for GlcNAc-treated mice compared to untreated controls. Data shown are mean ± SEM, n = 10, analysed by one-way ANOVA followed by Dunnett’s multiple comparison test compared with vehicle control, with significant differences represented vertically as *p < 0.05, **p < 0.01 and ***p < 0.001. **(G)** Time-course and relative abundance of serum ^13^C_6_-GlcNAc and Glc-D_7_ in mice gavaged with bolus administration of ^13^C_6_-GlcNAc alone or together with Glc-D_7_, n = 1. **(H)** At 180 min following gavage with ^13^C_6_-GlcNAc, UDP-^13^C_6_-GlcNAc was detected as a strong peak in different mouse tissues, with arbitrary units normalized to tissue weight.

To identify dietary conditions where a modifying effect of GlcNAc supplementation on body-weight could be detected, we supplied three groups of young male C57BL/6 mice with diets containing 4%, 9% and 22% fat content, whereas 6% fat is typically the standard diet for maintenance. As expected, dietary fat content correlated with increased body-weight and with decreased RER (Fig. 1A,B). Rates of weight-gain differed significantly in mice on different fat diets, with near cessation of growth on 4% diet, while weight-gain was progressive on 9% and 22% diet. The 4% and 9% fat diets were selected for further experiments, as both left capacity for more weight-gain and represented diverse base-line dietary conditions.

To explore dosage, GlcNAc at 0.5, 5.0 and 15 mg/ml (80–2,500 mg/kg/day) was continuously provided in drinking water to weight and age-matched male mice on 4% fat diet post-weaning. Body-weight was measured weekly and hepatic metabolites by liquid-chromatography tandem mass-spectrometry (LC-MS/MS) at 90 days of treatment. Body-weight during this juvenile period of rapid growth was increased in 5.0 and 15 mg/ml GlcNAc-treated mice (Fig. 1C). Metabolite profiling of liver by targeted LC-MS/MS revealed, using principal component analysis (PCA), a shift in relative metabolite abundance with GlcNAc dosage, moving away from the cluster of untreated controls (Fig. 1D). More specifically, tricarboxylic acid (TCA) cycle metabolites increased (Fig. S1A), while essential amino acids Thr, Trp, Phe and Ile/Leu decreased (Fig. 1E). The ratio of NADH to NAD+ was elevated, consistent with increased catabolism supplying oxidative phosphorylation (Fig. S1B). Furthermore, intermediates in hepatic glycolysis and gluconeogenesis were increased with oral GlcNAc, including Glc-6P, Fru-6P, phosphoenolpyruvic acid (PEP), lactate, glycerol and glycerol-3P (Fig.1F). Importantly, GlcNAc at 0.5 mg/ml or greater in the drinking water increased hepatic GlcNAc-P and UDP-GlcNAc by ~25%. (Fig. 1F).

A gavage was performed with heavy-GlcNAc (^13^C_6_-GlcNAc) to determine whether ingested GlcNAc enters the circulation and contributes directly to the UDP-GlcNAc pool in tissues. Serum ^13^C_6_-GlcNAc peaked at 30 min and was then completely cleared from the circulation by 2 to 3 h (Fig. 1G), at which time UDP-^13^C_6_-GlcNAc was detected in liver, kidney and spleen (Fig. 1H). After normalizing to tissue weight, UDP-^13^C_6_-GlcNAc was present in the liver at 44 U/mg, with lower levels in kidney (26 U/mg) and spleen (11 U/mg). GlcNAc was also co-administered with heavy-glucose (D_7_-Glc), which showed maximum levels in circulation at ~15 min, indicating earlier absorption than ^13^C_6_-GlcNAc (Fig. 1G). Nonetheless, the similarity in pharmacokinetics displayed by Glc-D_7_ and ^13^C_6_-GlcNAc suggests active transport of GlcNAc in the upper gastrointestinal tract and translocation into systemic circulation. The gavage experiment suggested GlcNAc uptake is rapid and may largely precede transit to the large intestine where the bulk of the gut microbiome resides. However, given the potential influence of the gut microbiome on obesity^28^, we examined the impact of oral GlcNAc on relative abundance of gut bacteria at the phylum level, to determine whether early changes might precede longer-term weight-gain observed in GlcNAc-treated mice. No significant differences were observed after 2 weeks of GlcNAc supplementation compared to untreated controls (Fig. S1C). Importantly, GlcNAc treatment did not alter the relative abundance of *Bacteroidetes* or *Firmicutes* (Fig. S1D,E), two phyla that have been associated with obesity and energy homeostasis in mice and humans^28^.

### Oral GlcNAc Increases Body-Weight Without Increasing Food Consumption

**Figure 2:**
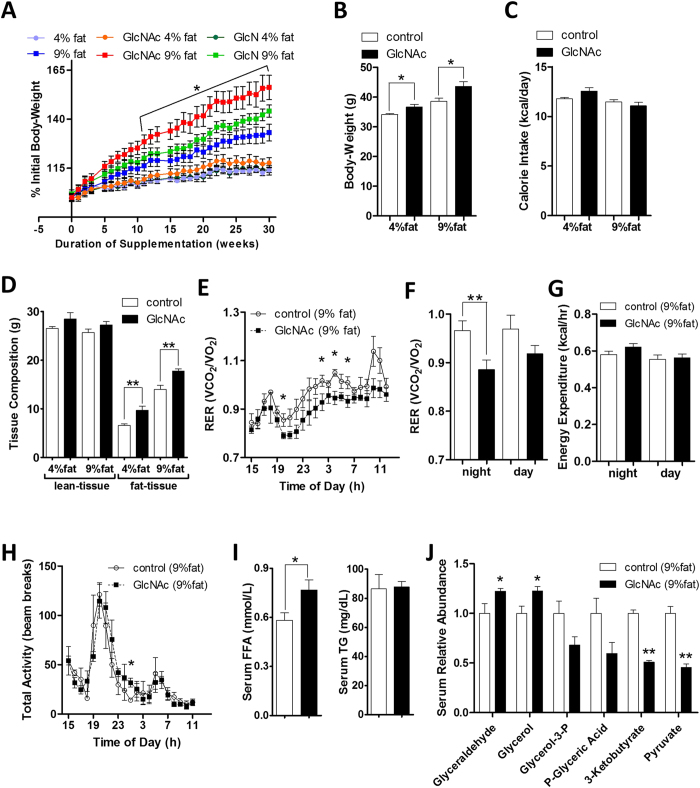
Oral GlcNAc promotes weight-gain and lipid accumulation in adult mice. **(A)** Change in body-weight for wild-type C57BL/6 male mice on 4% or 9% fat diet with GlcNAc or GlcN supplemented drinking water at 0.5 mg/ml. Data shown are mean ± SEM, n = 10, analyzed by 2-tailed unpaired Student’s *t*-test, with significant differences indicated as *p < 0.05 for 9% fat control versus 9% fat on GlcNAc. **(B)** Body-weight and **(C)** calorie-intake per mouse per day following 21 weeks of GlcNAc treatment. **(D)** Lean and fat tissue composition measured by dual-energy X-ray absorptiometry (DEXA). Data shown are mean ± SEM, n = 10, *p < 0.05 and **p < 0.01 GlcNAc-treated versus control on either 4% or 9% fat diet. **(E)** Respiratory Exchange Ratio (RER = VCO_2_/VO_2_) over 20 h period, **(F)** RER quantification by night and day, **(G)** energy expenditure and **(H)** total activity in mice supplemented with oral GlcNAc on 9% fat diet. Data shown are mean ± SEM, n = 5, *p < 0.05 and **p <  < 0.01. **(I)** Serum free fatty-acids (FFA) and triglycerides (TG), and **(J)** serum metabolite changes in mice on 9% fat diet and supplemented with 0.5 mg/ml oral GlcNAc for 90 days. Data shown are mean and error bars represent ± SEM, n = 5, *p < 0.05 and **p < 0.01 GlcNAc-treated versus control with 2-tailed, unpaired Student’s *t*-test.

We adopted 0.5 mg/ml of GlcNAc to test for interaction with a fat-enriched diet in mature adult mice. GlcNAc was initiated at about 3 months of age in weight-matched wild-type C57BL/6 male mice maintained on either 4% or 9% fat diets, and continued for 30 weeks (Fig. 2A). Mice were also treated with glucosamine (GlcN), a related amino-sugar which is transported efficiently via hexose transporters^29^, followed by two possible fates, either N-acetylation by Gnpnat1/Gna1 to GlcNAc-6P or deamination by Gnpda1 to Fru-6P and catabolism in glycolysis^30^. GlcNAc-treated mice on 9% fat diet displayed significantly increased weight-gain compared to 9% fat diet alone or treatment with GlcN (Fig. 2A). The insignificant effect of GlcN is consistent with its deamination and loss to the UDP-GlcNAc pool^23^. After 20 weeks of GlcNAc treatment, the mice displayed on average 13% and 19% increase in body-weight on 4% and 9% fat diet respectively (Fig. 2B), without any discernible increase in daily calorie-intake per mouse (Fig. 2C). At 30 weeks, GlcNAc-treated mice weighed 10% more on 4% fat diet and 16% more on 9% fat diet than their control counterparts (Fig. S2A). GlcNAc-treated mice had similar lean-tissue mass, but displayed increased fat content on both diets, as determined by DEXA (Fig. 2D). The epididymal fat-pads were increased with GlcNAc treatment by 46% and 12% on 4% and 9% fat diets respectively (Fig. S2B). Oral GlcNAc with 9% fat diet resulted in a 42% increase in liver weight, a 22% increase after correcting for body-weight. The 0.5 mg/ml GlcNAc dosage (translating into 40–80 mg/kg per day) was equivalent to less than 0.1% of total weight of daily food intake per mouse, an insignificant source of calories, suggesting the effects on metabolism depend on GlcNAc conversion to UDP-GlcNAc and protein glycosylation.

### Oral GlcNAc Does Not Alter Physical Activity or Energy Expenditure

Open circuit indirect calorimetry was used to estimate whole-body O_2_ consumption and CO_2_ production, while the activity of mice was measured by infrared photocells. RER was calculated from O_2_ consumed and CO_2_ produced (Fig. 2E, S2C,D), and provides a measure of nutrients oxidized that ranges from 0.7 for oxidation of pure fats to 1.0 for oxidation of pure carbohydrates. Decreased RER in GlcNAc supplemented mice on 9% fat diet suggests more oxidation of fat for energy generation compared to mice on 9% fat diet alone, especially at night when mice are most active (Fig. 2F). GlcNAc-treated and untreated mice on 9% fat diet were indistinguishable in whole-body energy expenditure and total activity (Fig. 2G,H). Serum triglycerides (TG) were unchanged, while free fatty-acids (FFA) were increased in GlcNAc-treated mice (Fig. 2I), suggesting enhanced lipid catabolism as well as accumulation, a conclusion also supported by the increase in serum glycerol and glyceraldehyde; whereas, pyruvate, glycerol-3-phosphate, phosphoglyceric acid and ketone-body 3-ketobutyrate were decreased (Fig. 2J).

### Oral GlcNAc Increases Lipid Accumulation

**Figure 3:**
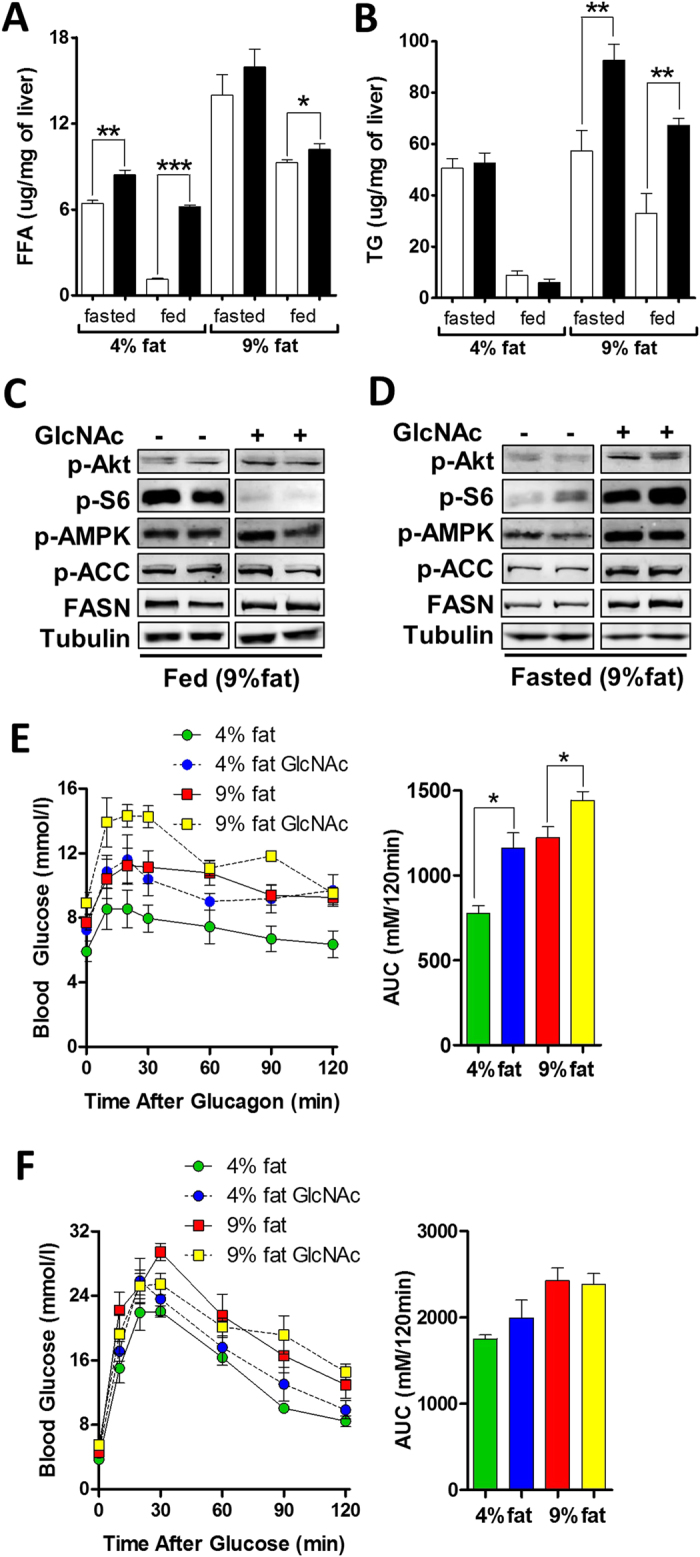
Oral GlcNAc alters liver metabolism. **(A)** Liver free fatty-acids (FFA) and **(B)** triglycerides (TG) in wild-type C57BL/6 male mice on 4% or 9% fat diet over 30 weeks on oral GlcNAc at 0.5 mg/ml. Data shown are mean ± SEM, n = 5, *p < 0.05, **p < 0.01 and ***p < 0.001 GlcNAc-treated versus control in fasted or fed state with 2-tailed, unpaired Student’s *t*-test. Immunoblot analysis of metabolic signaling pathways, with fatty-acid synthase (FASN) and phosphorylated forms of Akt kinase, ribosomal protein S6 (S6), AMP-Activated Protein Kinase (AMPK-α) and Acetyl-CoA Carboxylates Kinase (ACC) in liver lysates from mice maintained on 9% fat diet and supplemented with GlcNAc for 30 weeks in *ad libitum* fed **(C)** or 18 h fasted **(D)** states. **(E)** Intraperitoneal glucagon tolerance test and **(F)** intraperitoneal glucose tolerance test, with area under the curve (AUC) quantification in mice supplemented with 0.5 mg/ml oral GlcNAc for 23 weeks. Data shown are mean ± SEM, n = 5, *p < 0.05.

GlcNAc increased hepatic FFA on 4% fat diet in fasted and fed mice, and to a lesser degree on 9% fat, where FFA in controls were already high (Fig. 3A). Hepatic TG levels in fasted and fed mice were increased by GlcNAc on 9% fat diet (Fig. 3B). Serum alanine aminotransferase (ALT), a non-specific marker of liver damage was unchanged (Table 1). In fed mice GlcNAc treatment on a 9% fat diet showed reduced hepatic phosphorylation of ribosomal protein S6 (pS6) (Fig. 3C), suggesting reduced mTORC1 activity and increased autophagy/macrolipophagy^31^. Phosphorylated AMP-activated protein kinase (p-AMPK-α) and its downstream target Ac-CoA carboxylase kinase (p-ACC), indicators of lower energy charge, reduced fatty-acid synthesis and increased fatty-acid oxidation^32^, were unchanged by GlcNAc in livers of fed mice, while fatty-acid synthase (FASN), the key enzyme in *de novo* lipogenesis was somewhat elevated (Fig. 3C). With 18 h fast, hepatic p-S6, p-Akt, p-AMPK-α, p-ACC and FASN were increased in GlcNAc-treated mice relative to controls (Fig. 3D), and comparable to livers of untreated fed mice, suggesting a delayed fasting response. Indeed, liver glycogen levels remained higher in fasted GlcNAc-treated mice (Fig. S2E,F). Collectively, these results suggest that GlcNAc enhances the efficiency of nutrient uptake during fasting, and/or provides extra support for hepatic anabolic metabolism from muscle and adipose tissues to delay the fasting response. The effects of oral GlcNAc on blood glucose and serum levels of insulin, glucagon and leptin during fasting were consistent with this interpretation (Table 1). The ratio of circulating insulin to glucagon was indistinguishable between fasted and fed GlcNAc-treated mice, while the ratio changed 12-fold in untreated mice (Table 1). Oral GlcNAc increased circulating serum leptin in both fasted and fed states (Table 1), implying satiety and reduced appetite, and is consistent with GlcNAc-supplemented mice not consuming more calories (Fig. 2C).

Mgat5 activity enhances glucagon receptor sensitivity in cell culture and in mice^13^. Indeed, GlcNAc-treated mice displayed enhanced sensitivity to an injection of glucagon (Fig. 3E), indicated by increased release of hepatic glucose, while glucose tolerance was not affected (Fig. 3F). The hypersensitivity of glucagon receptor should place a higher demand on amino acids and other gluconeogenic precursors, the source of carbon for liver gluconeogenesis, as well as insulin secretion to clear the excess hepatic glucose released into circulation. Consistent with this, serum lactate and amino acids Gln, Phe, Tyr, Leu and Ile were elevated after 30 weeks of oral GlcNAc (Fig. S2G), possibly a result of autophagy in muscles to support anabolic metabolism in the liver. Serum Gln, Phe, Ile, Tyr and Leu are associated with human obesity and a higher risk of diabetes^33^.

### Oral GlcNAc Increases Complex N-Glycan Branching in Liver Glycoproteins

Glycopeptides were prepared from liver tissue of control and GlcNAc-treated mice, and differentially labelled with light and heavy stable-isotope dimethyl reagents respectively. Light and heavy labelled glycopeptides were mixed prior to mass-spectrometry analysis. Intact glycopeptides and their deglycosylated counterparts, obtained by treatment with peptide-N-glycosidase F were analyzed by LC–MS/MS^34^. The combined analyses identified N-glycosylated sites in liver glycoproteins and the composition of N-glycans at each site^34^. Specific sites identified by peptide sequence were compared for GlcNAc content in control and GlcNAc-treated mice. Mice supplemented with GlcNAc on 4% and 9% fat diets, in both fed and fasted conditions, displayed significantly increased global GlcNAc content in liver N-glycans, which based on the biosynthesis pathway must be attributed to increased N-glycan branching (Table 2). As a specific example, glycopeptides with Asn89 specific N-glycosylation from the single transmembrane pass glycoprotein carcinoembryonic antigen related cell adhesion molecule 1 (CEACAM1 or CD66a) were analyzed in detail. After normalizing with the GlcNAc-treated to control ratio of deglycosylated peptides (Fig. S3A,B), the tri-antennary N-glycans on Asn89 of CEACAM1 were 13-fold more abundant in livers from GlcNAc-supplemented mice (Fig. S3C–D). In contrast, hybrid and complex bi-antennary N-glycans displayed a normalized GlcNAc-treated to control ratio of 0.72 and 0.70 respectively (Fig. S3E–H), a concomitant decrease in less branched N-glycans. Taken together these results suggest oral GlcNAc entered systemic circulation and was taken up by the liver, where it increased hepatic UDP-GlcNAc supply to tri-antennary N-glycan branching. Tetra-antennary N-glycans were not detected, consistent with previous reports that hepatic Mgat4a/b expression, activity and associated N-glycan structures are very low^12^,^35^.

### GlcNAc Increases Nutrient Uptake in Support of Lipid Accumulation

**Figure 4:**
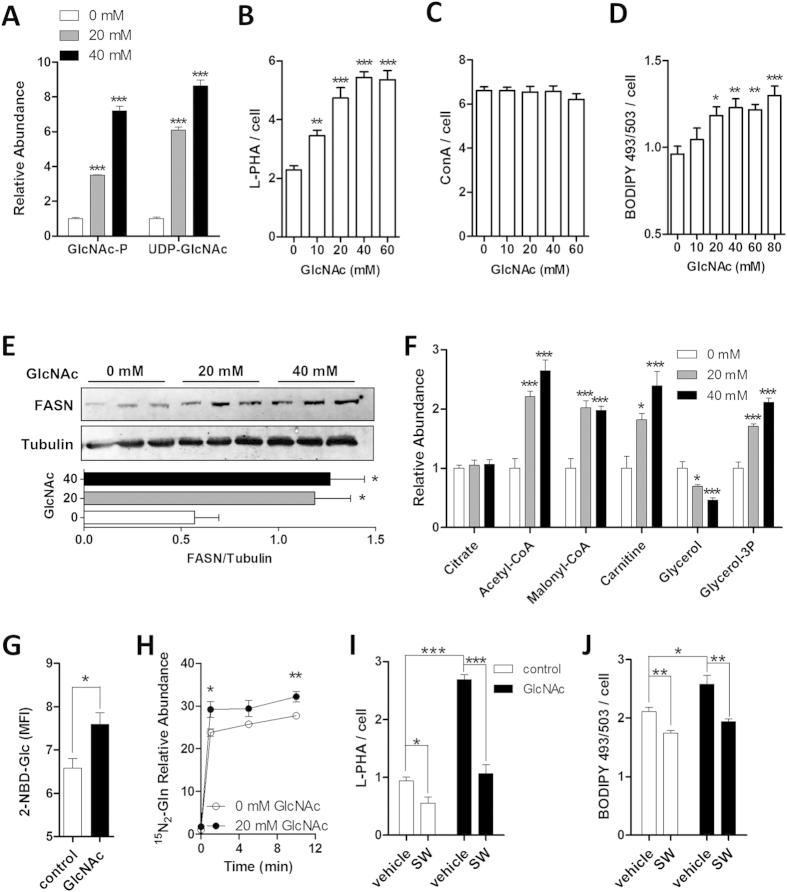
GlcNAc increases UDP-GlcNAc, β-1,6-GlcNAc branched N-glycans and lipid accumulation in AML12 cells. **(A)** Fold changes of distal HBP metabolites upon GlcNAc treatment. **(B)** Analysis of Mgat5-dependent N-glycan branching on cell surface glycoproteins quantified with Alexa-488-conjugated lectin L-PHA. **(C)** Analysis of oligomannose-type and hybrid-type N-glycans on cell surface glycoproteins with Alexa-488-conjugated lectin ConA. **(D)** Lipid droplet content, quantified microscopically with lipophilic fluorescent probe BODIPY 493/503. **(E)** Western blot analysis of enzyme fatty-acid synthase (FASN) and loading control tubulin, used for relative quantification of immunoblot. **(F)** Fold change in specific metabolites involved in fat metabolism, normalized to cell number. **(G)** Glucose uptake in cells treated with GlcNAc for 20h, grown in the presence of fluorescent Glc-analog 2-NBD-Glc for 1h, and quantified using flow cytometry as mean fluorescent intensity (MFI). **(H)** Analysis of heavy-isotope dual-labelled Gln uptake in cells pretreated with GlcNAc, pulsed with ^15^N_2_-Gln for designated times, and quantified using mass spectrometry. **(I)** Tri- and tetra-antennary Mgat5-modified N-glycans and **(J)** lipid droplet content in the absence and presence of GlcNAc and/or Swainsonine (SW), quantified microscopically with Alexa-488-conjugated lectin L-PHA or BODIPY 493/503. Data shown are mean ± SEM, analyzed by 2-tailed unpaired Student’s t-test or one-way ANOVA followed by Dunnett’s multiple comparison test compared with vehicle control (A-F), with significant differences represented as *p < 0.05, **p < 0.01 and ***p < 0.001.

In Hek293 cells GlcNAc and induced overexpression of Mgat5 has been shown to promote N-glycan branching, nutrient uptake, increased intracellular metabolite levels, and cell growth in low glucose and glutamine conditions^22^. We explored whether GlcNAc also promotes nutrient uptake and lipid accumulation in AML12 cells, an immortal mouse hepatocyte cell line, in an N-glycan branching-dependent manner. Supplementation with GlcNAc increased GlcNAc-P over 3-fold and UDP-GlcNAc 6-fold (Fig. 4A), as well as binding of L-PHA, a lectin probe for Mgat5-modified complex-type tri- and tetra-antennary branched N-glycans (Fig. 4B). Binding of Concanavalin-A (ConA), a lectin that binds oligomannose- and hybrid-type N-glycans was unchanged (Fig. 4C). This indicates that N-glycosylation and early processing in the ER were not altered by increasing cellular UDP-GlcNAc, whereas N-glycan branching was sensitive to UDP-GlcNAc levels. Lipid droplet content and FASN level increased with GlcNAc treatment in a dose-dependent manner (Fig. 4D,E). Furthermore, metabolites involved in fat metabolism such as citrate, Ac-CoA, malonyl-CoA, carnitine and glycerol-3P increased, while glycerol, the immediate precursor for glycerol-3P was depleted (Fig. 4F). As metabolites that supply *de novo* lipogenesis, the uptake of fluorescent glucose-analog (2-NBD-Glc) and dual-isotope labelled glutamine (^15^N_2_-Gln) was increased with GlcNAc treatment in AML12 cells (Fig. 4G,H). Swainsonine (SW), an inhibitor of Golgi α-mannosidase II, blocks N-glycan-mediated branching by Mgat2, Mgat4 and Mgat5^15^. SW reduced GlcNAc-dependent increase in N-glycan branching, and importantly blocked GlcNAc-induced increase in lipid droplet accumulation in AML12 cells (Fig. 4I,J). GlcNAc also enhanced ^15^N_2_-Gln uptake in the immortal epithelial HeLa cells, along with increasing lipid accumulation, UDP-GlcNAc levels, and Mgat5-modified N-glycans (Fig. S4A–D). Similarly, 3T3-L1 fibroblasts differentiated into adipocytes exhibited a GlcNAc dose-dependent increase in Mgat5-mediated N-glycan branching, fatty-acid uptake and lipid accumulation (Fig. S4E–G). Thus, different cell lines respond to GlcNAc with elevation of UDP-GlcNAc, N-glycan branching, nutrient uptake and lipid accumulation.

### Oral GlcNAc Partially Restores Anabolic Metabolism in Mgat5^−/−^ Mice

**Figure 5:**
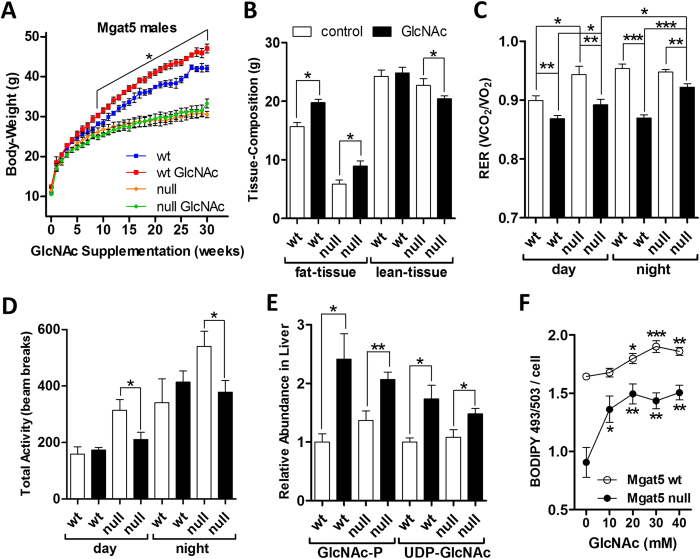
Oral GlcNAc partially rescues lipid storage in Mgat5^−/−^ mice. **(A)** Change in body-weight for Mgat5^+/+^ and Mgat5^−/−^ male mice on 9% fat diet supplemented with 0.5 mg/ml oral GlcNAc in drinking water for 30 weeks. Data shown are mean ± SEM, n = 4–5, with significant difference indicated as *p < 0.05 for wt control versus wt on GlcNAc. **(B)** Fat and lean tissue mass content, measured by EchoMRI. **(C)** Diurnal and nocturnal respiratory exchange ratio (RER) and **(D)** total activity (locomotion, grooming and rearing on hind legs) during the same time period. **(E)** Relative abundance of liver GlcNAc-P and UDP-GlcNAc determined in the same cohort of mice and expressed as fold change. Data shown in panels above are mean ± SEM, n = 4–5, with statistical significance indicated as *p < 0.05 and **p < 0.01 for GlcNAc-treated versus untreated control groups of the same genotype with 2-tailed, unpaired Student’s t-test. **(F)** Lipid droplet content in primary hepatocytes derived from 3 month old Mgat5^+/+^ and Mgat5^−/−^ mice and cultured overnight with exogenous GlcNAc supplementation. Lipid was quantified with the lipophilic fluorescent probe BODIPY 493/503. Data shown are mean ± SEM, n = 4, analyzed by one-way ANOVA followed by Dunnett’s multiple comparison test compared to 0 mM GlcNAc control of respective genotype, with significant differences indicated as *p < 0.05, **p < 0.01 and ***p < 0.001.

GlcNAc-supplementation in Mgat5^−/−^ mammary tumor cells has been shown to rescue a deficiency in cell surface retention of TGF-β and EGF receptors^15^,^36^. The rescue is due to compensating increases in the activity of the remaining Mgat branching enzymes driven by increased UDP-GlcNAc. The N-acetyllactosamine branches are additive in rescuing affinities for galectins that regulate glycoprotein dynamics at the cell surface^15^. Mgat5^−/−^ mice display reduced body-weight and fat content^19^, a phenotype opposite to that observed with GlcNAc-supplementation in wild-type mice. Therefore, we attempted to rescue body-weight in Mgat5^−/−^ mice with oral GlcNAc on 9% fat diet, which had no significant effect by this measure (Fig. 5A). However, fat tissue increased by 53% compared to only 26% increase in Mgat5^+/+^ mice, but was offset by an 11% decrease in lean-tissue mass in Mgat5^−/−^ male mice (Fig. 5B). Similar results for body-weight and tissue composition were observed in Mgat5^+/+^ and Mgat5^−/−^ female mice on 9% fat diet (Fig. S5A,B). Serum leptin was lower in Mgat5^−/−^ than Mgat5^+/+^ mice, and GlcNAc increased its levels in Mgat5^+/+^ but did not reach significance in Mgat5^−/−^ mice (Fig. S5D). Oral GlcNAc lowered RER in both genotypes (Fig. 5C), but significantly less in Mgat5^−/−^ mice at night. GlcNAc-treated Mgat5^−/−^ mice were less active than untreated Mgat5^−/−^ mice (Fig. 5D), possibly a consequence of the relative decrease in muscle mass and increase in fat tissue. Hepatic GlcNAc-P and UDP-GlcNAc were elevated by oral GlcNAc in both genotypes (Fig. 5E). Primary hepatocytes from young Mgat5^−/−^ mice cultured overnight displayed a 45% lower lipid droplet content than hepatocytes from Mgat5^+/+^ counterparts (Fig. 5F). GlcNAc increased lipid droplet content in Mgat5^−/−^ hepatocytes to levels approaching that of untreated Mgat5^+/+^ hepatocytes (Fig. 5F). Collectively, GlcNAc increased fat accumulation as well as fat oxidation in Mgat5^−/−^ mice, consistent with the model of functional redundancy in N-glycan branches supported by UDP-GlcNAc supply. However, either the GlcNAc dosage was limiting and/or Mgat5 modified N-glycans are required for the normal balance of lean to fat body tissue composition. Interestingly, Mgat5-dependent branching decreases with aging^19^, and may contribute to the well-known increase in insulin resistance and increased fat content in liver and muscle with age^37^.

## Discussion

Here we have examined the effects of oral GlcNAc-supplementation on mouse physiology and metabolism. LC-MS/MS analysis revealed that GlcNAc ingested orally is rapidly absorbed, enters systemic circulation, and is used by tissues in HBP to increase intracellular UDP-GlcNAc pool. In the post-weaning rapid-growth phase mice on a 4% fat diet showed more weight-gain with oral GlcNAc, as well as increased hepatic levels of HBP, glycolytic, gluconeogenic and TCA metabolites, and reduced levels of most amino acids. With prolonged oral GlcNAc weight-gain was enhanced, with greater effect on 9% compared to 4% fat diet, evidence of a clear interaction between GlcNAc and calorie enriched fat diet. GlcN at a similar dosage did not significantly increase body-weight, suggesting a less potent contribution to UDP-GlcNAc and down-stream effectors. Parameters that were not significantly altered by oral GlcNAc included daily calorie-intake, total activity, energy expenditure, and the gut microbiome. Thus, GlcNAc-treated mice are not indolent or lethargic but rather utilize equivalent calories more efficiently, as determined by increased conversion to body-mass and fat content. This apparent gain in efficiency may include enhanced absorption (uptake) of nutrients in the gut and from systemic circulation, without violating the first law of thermodynamics. Although GlcNAc as a monosaccharide did not alter the gut microbiota herein, perhaps dietary polymers composed of GlcNAc such as chitin may do so in the large intestine, since glycosidase diversity varies between microbiota populations along the gut^38^. As such, the microbiota may regulate release of GlcNAc from intestinal mucins or dietary polysaccharides, which in turn might affect its species distribution.

**Figure 6:**
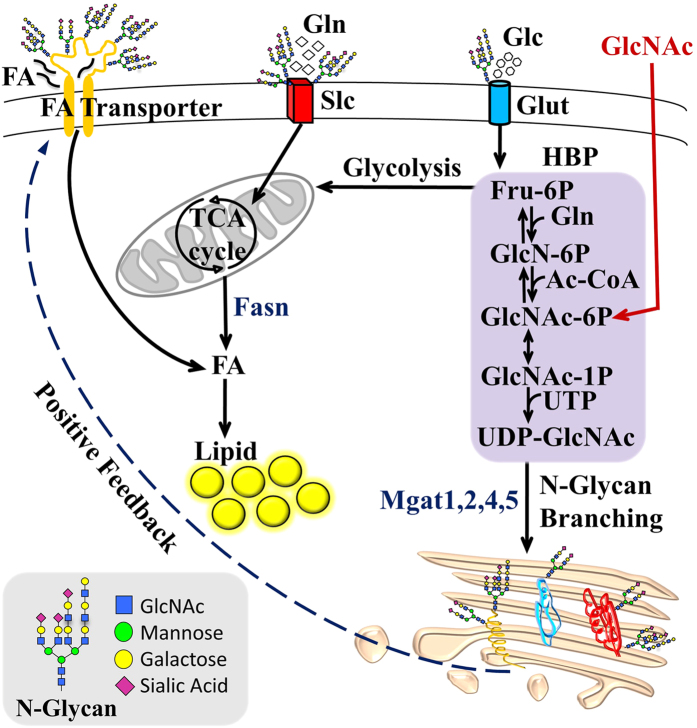
Proposed model of HBP and N-glycan branching dependent regulation of cellular metabolism. GlcNAc salvaged by HBP increases UDP-GlcNAc, the substrate for branching N-acetylglucosaminyltransferases (Mgat1,2,4,5), which modify glycoproteins trafficking through the Golgi *en route* to the cell surface^20^. *K*_*m*_ values for UDP-GlcNAc decline from Mgat1, Mgat2, Mgat4 to Mgat5, thus biosynthesis of tri- and tetra-antennary N-glycans is sensitive to UDP-GlcNAc levels^20^. Titration of N-glycan branching via UDP-GlcNAc increases the affinity of glycoproteins for galectins, which cross-link and oppose loss of receptors and transporters to endocytosis^20^. This may stabilize cell surface residency and thereby activity of glucose (Glc), glutamine (Gln) and fatty-acid (FA) transporters (Glut, Slc and FA transporters, respectively). More nutrient uptake contributes to increase in FA synthesis and lipid accumulation via Fasn. A positive-feedback loop is formed by increasing uptake and flux of Glc, Gln and Ac-CoA through *de novo* HBP to UDP-GlcNAc and Golgi N-glycan branching on glycoprotein transporters and receptors.

Oral GlcNAc-supplementation increases hepatic UDP-GlcNAc and overall GlcNAc content in glycoprotein N-glycans, consistent with more highly branched cell surface N-glycans. In cultured cells GlcNAc increased the UDP-GlcNAc pool and Mgat5-dependent N-glycan branching, enhanced uptake of glucose, glutamine and fatty-acid, and increased lipid accumulation. These results suggest a potential mechanism for metabolic reprogramming by HBP and Golgi N-glycan branching pathway. GlcNAc driven HBP flux promotes N-glycan branching and thereby retention of receptors^15^ and transporters^22^ that promote nutrient uptake and reprogram cellular metabolism, leading to lipid accumulation in replete conditions (Fig. 6). The hepatic phenotype of GlcNAc-treated mice on 9% fat diet suggests metabolic stress and ectopic accumulation of lipids, leading to hepatic steatosis. Oral GlcNAc delayed or altered the normal fasting response. The atypical response to fasting, as revealed by hormonal profile, hepatic glycogen content, and AMPK, Akt and mTORC1 signaling suggests a continued abundance of nutrients and/or hyperinsulinaemia. Following an 18 h fast, liver of GlcNAc-treated mice maintained on 9% fat diet revealed a dramatic elevation in S6 phosphorylation, a marker of mTORC1 pathway activity^39^. Since hepatic mTORC1 activity negatively regulates autophagy^31^ and ketone body production as an energy source^40^, this observation suggests decreased autophagy/macrolipophagy and ketogenesis in fasted GlcNAc-treated mice. Oral GlcNAc was associated with elevated fasting levels of blood Glc, Gln, Ile/Leu and lactate, as well as hormones leptin and insulin, which positively correlate with body-weight and energy stores^41^. Leptin also stimulates fatty-acid oxidation in non-adipose tissues, so as to minimize ectopic lipid accumulation and protect against lipotoxicity^41^. The insulin to glucagon ratio was abnormally low in fed and high in fasted GlcNAc-treated mice on 9% fat diet, with no change between fed and fasted states compared to the normal 12-fold decrease observed with fasting in control mice.

Genes in HBP have been implicated in lipid accumulation and characterized as promoting a thrifty phenotype^7^. Polymorphisms in Gfpt are associated with obesity in men^42^ and fat content in swine^43^. Hepatic overexpression of Gfpt in mice resulted in excess synthesis of fatty-acids and triglycerides, and greater weight-gain compared to non-transgenic littermates^4^. Gfpt overexpression in HepG2 liver cells increased transcript levels of lipogenic genes Fasn, Acc and Srebp-1^44^. Genetic or pharmacological suppression of Gfpt inhibited lipogenesis in HepG2^44^, murine 3T3-L1 adipocytes^45^, and in human visceral adipocytes^46^. In the red flour beetle down-regulation of UDP-GlcNAc pyrophosphorylase (Uap1) resulted in depletion of fat-body tissue^47^. Hepatic Uap1 transcript level was more highly expressed in cattle bred for meat than in dairy breeds^48^. Increased liver expression of Slc35b4, a Golgi UDP-GlcNAc antiporter, was identified as a quantitative trait locus associated with high-fat diet-induced obesity, insulin resistance and gluconeogenesis in mice^49^. Slc35b4 was also identified as a candidate gene for obesity in humans^50^. Increased expression of Slc35b4 could provide more effective UDP-GlcNAc transport into Golgi, increasing its supply to N-glycan branching pathway, where Mgat5 is most sensitive to its concentration^20^. Furthermore, a recent report suggests that UDP-GlcNAc antiporter Slc35a3 forms a complex with Mgat5 enzyme in the Golgi membrane, and augments its catalytic activity by proximity^51^. Indeed, cells deficient in Slc35a3 displayed reduced levels of tri- and tetra-antennary N-glycans^51^.

In cultured mammalian cells GlcNAc contributes exclusively to UDP-GlcNAc, and does not appear to enter glycolysis, TCA or the pentose phosphate pathway^23^. UDP-GlcNAc supply to the Golgi N-glycan branching pathway and/or Mgat5 overexpression promotes glutamine and essential amino acid uptake in cultured cells under limiting nutrient conditions^22^. These and our current results suggest HBP and N-glycan branching interact descriptively as a thrifty phenotype^52^. Moreover, Mgat5 transgenic mice displayed increased body-weight and liver to body-weight ratio^53^, along with increased hepatic lipogenesis characterized by elevated Fasn, Acc and Srebp-1 gene expression^54^. Conversely, Mgat5^−/−^ mice are resistant to weight-gain on 9% fat diet, and display hypersensitivity to fasting with greater glycogen depletion and hypoglycemia^19^, essentially an opposing phenotype compared to GlcNAc-supplemented wild-type mice in the present study. GlcNAc restored fat accumulation in Mgat5^−/−^ mice and primary hepatocytes, consistent with a redundancy model where increasing UDP-GlcNAc drives compensating increases in N-glycan branching by other Mgat enzymes^15^,^55^. However, Mgat5^−/−^ mice treated with GlcNAc did not fully recover normal body-weight and displayed lower lean-tissue mass, suggesting either dosage of GlcNAc was not sufficient to completely rescue both tissue types, and/or Mgat5 plays a critical role in balancing fat and lean tissue with aging.

Early aging in Mgat5^−/−^ mice is associated with an imbalance between TGF-β and growth factor signaling, resulting in premature loss of muscle satellite cells and osteoprogenitor bone-marrow cells^19^. Positive feedback between metabolism, HBP and N-glycan branching may play a role in maintaining muscle satellite cells^19^. GlcNAc-supplementation also rescued glucagon receptor sensitivity in Mgat5^−/−^ primary hepatocytes and *in vivo* in mice^13^, and in this study enhanced its sensitivity in wild-type mice. However, the fine tuning of satellite cell regeneration and glucagon receptor sensitivity may require different proportions or levels of HBP, Mgat5 and N-glycan branching activity. Most solute transporters are N-glycosylated and further work is required to determine which ones are regulated by N-glycan branching. However it seems likely that hepatic amino acid uptake and catabolism, coupled with glucagon-driven gluconeogenesis is likely to play a major role in the phenotypes observed in GlcNAc-treated mice. Finally, it will be important to consider the interaction of UDP-GlcNAc as a substrate in multiple protein glycosylation pathways, to understand the full impact on metabolism.

As a practical consideration, the sources and amounts of GlcNAc in our diet are unknown. For example, chitin is a long-chain polymer of GlcNAcβ1–4 found widely in nature and used as a food and feed additive^56^, which is obscured on labelling as carbohydrates, sugar or fibre. Dietary forms of GlcNAc may interact with gene polymorphisms in HBP and Golgi N-glycan branching pathway^57^, playing a role in the obesity epidemic. On the other hand, our data also suggests that GlcNAc-supplementation may benefit individuals on a suboptimal diet or with medical conditions where nutrient absorption is compromised.

## Methods

### Chemicals and Materials

N-acetylglucosamine (GlcNAc) was obtained as “Ultimate Glucosamine” (Wellesley Therapeutics, Toronto, Ontario, Canada). Metabolite standards and reagents were obtained from Sigma Chemicals (St. Louis, MO) with minimal purity of 98%. Glucose-D_7_ (Glc-D_7_) and ^15^N_2_-L-Glutamine were purchased from Cambridge Isotope Laboratories Inc. (Andover, MA), ^13^C_6_-GlcNAc from Omicron Biochemicals Inc. (South Bend, IN). All organic solvents and water used in sample and LC/MS mobile phase preparation were LC/MS grade and obtained from Fisher Scientific (Fair Lawn, NJ). Antibodies to phospho-Thr172 AMPK-α, phospho-Ser79 ACC, phospho-Ser235/236 S6, and phospho-Ser473 AKT were purchased from Cell Signaling Technology. Antibody to Tubulin was purchased from Sigma-Aldrich, and FASN from BD Scientific. PhosSTOP Phosphatase Inhibitor Cocktail and Complete Protease Cocktail were purchased from Roche. PVDF membrane (Immuno-Blot, 0.2 μm, 7.0 × 8.5 cm) purchased from Bio-Rad (Hercules, CA). Alexa Fluor-488 conjugated lectins Concanavalin A (ConA) and Leucoagglutinin *P. vulgaris* (L-PHA), BODIPY 493/503 (4,4-Difluoro-1,3,5,7,8-Pentamethyl-4-Bora-3a,4a-Diaza-s-Indacene), and 2-NBD-Glucose (2-(N-(7-Nitrobenz-2-oxa-1,3-diazol-4-yl)Amino)-2-Deoxyglucose) were purchased from Invitrogen (Carlsbad, CA). QBT Fatty-Acid Uptake Assay Kit containing BODIPY-dodecanoic acid fluorescent fatty-acid analog was purchased from Molecular Devices (Sunnyvale CA). Mouse endocrine LINCOplex kit for insulin, glucagon and leptin hormones were purchased from Linco Research.

### Mice

Weight and age-matched young C57BL/6 male mice were used in GlcNAc-supplementation experiments. Mgat5^+/+^ and Mgat5^−/−^ mice, age and sex-matched littermates on the C57BL/6 background were used as described previously^13^,^19^. All mice were maintained in cages of up to 5 mice per cage, in a normal 12-h light/12-h dark cycle on either 4%, 9% or 22% fat food diet (Tekland rodent diet), with or without GlcNAc (0.5, 5.0 or 15 mg/ml) or GlcN (0.5 mg/ml) in drinking water, as indicated, for the specified duration of time. Bottles with drinking water containing GlcNAc or GlcN were changed twice weekly. Mice were euthanized using CO_2_ inhalation and dissections carried out rapidly to remove, weigh, and freeze liver tissue samples on dry-ice, and store in tubes at −80 °C until further analysis. All experiments using mice were conducted according to protocols and guidelines approved by the Toronto Centre for Phenogenomics animal care committee. To profile the bacterial gut microbiome 16S rRNA gene sequencing and analysis was performed on fecal samples from GlcNAc-treated mice before and after supplementation – see supplementary material for details.

### Phenotyping *in vivo*

Body-weight of mice was measured on a weekly basis. Daily food consumed was determined every 24 h over 10 days, and expressed as calorie-intake per mouse per day, at 21 weeks following start of GlcNAc-supplementation. Body composition, in terms of lean and fat tissue mass, was determined by dual-energy X-ray absorptiometry (DEXA) (PIXImus) or EchoMRI (Echo Medical Systems). Whole-body O_2_ consumption and CO_2_ production rates were recorded for 20 h with the use of an open-circuit indirect calorimeter (Oxymax Lab Animal Monitoring System, Columbus Instruments). Respiratory exchange ratio (RER) was calculated as molar ratio of VCO_2_ to VO_2_ for 5 mice per group, averaging the measurements for the light and dark cycle. The activity of mice in three spatial dimensions plus time was continuously measured during the same 20 h using infrared photocells attached to the metabolic cage during dark and light cycles. Total activity included ambulatory movement (locomotion), and body movement (grooming and rearing on hind legs). Water and food were available *ad libitum* in the metabolic chamber. To minimize the potential influence of circadian rhythms on experimental outcomes, standardized periods of fasting or experimental analyses were utilized. For intraperitoneal glucose tolerance test, mice were fasted for 18 h before intraperitoneal injection of 0.01 ml/g of body-weight of a glucose solution containing 150 mg/ml. For the glucagon tolerance test, mice were fasted for 5 h and injected intraperitoneally with a glucagon solution of 1.6 g/ml (0.01 ml/g of body-weight). Blood samples were drawn from the tail vein at regular time intervals over the course of 2 h, and blood glucose levels measured using Glucometer Elite blood glucose meter (Bayer, Toronto, Canada). Young male mice weighing 20 g were fasted for 4 h and orally gavaged with a bolus administration of ^13^C_6_-GlcNAc at 20 μg/g alone, or in combination with Glc-D_7_ at 50 μg/g. Blood from the tail vein was collected at regular time intervals during a 3 h time-course, kept at 4 °C, spun to recover the serum, and stored at −80 °C for further analysis of ^13^C_6_-GlcNAc or D_7_-Glc by targeted mass-spectrometry. At 180 min following oral gavage mice were sacrificed and liver, kidney and spleen removed and stored at −80 °C for further analysis of UDP-^13^C_6_-GlcNAc in tissue by targeted mass-spectrometry.

### Targeted metabolomics

Frozen liver tissue (~100 mg per sample) was pulverized using the CellCrusher^TM^ cryogenic tissue pulverizer under liquid nitrogen. The soluble polar metabolites were extracted by addition of 1 ml of ice-cold extraction solvent consisting of 40% acetonitrile, 40% methanol and 20% water, followed by vortexing for 30 sec, and vigorous shaking for 1 h at 4 °C in a ThermoMixer (Eppendorf, Germany). Following extraction, samples were spun down at 20,000xg for 10 min at 4 °C, and the supernatant transferred to fresh tubes to be evaporated to dryness in a CentriVap concentrator at 40 °C (Labconco, MO). The dry extract samples were stored at −80 °C for LC–MS/MS analysis. Metabolites were analyzed at the optimum polarity in MRM mode on electrospray ionization triple-quadrupole mass spectrometer (4000 QTRAP; ABSciex, Toronto, Canada) as previously described^2^. The LC-MS/MS system does not resolve isomers of hexose (glucose/galactose/mannose), n-acetyl-hexosamine (GlcNAc/GalNAc/ManNAc), or their phosphorylated or nucleotide-sugar forms. In the paper we refer to the Glc form of these isomers.

### Site-specific glycopeptide analysis

Frozen mouse liver tissue was cut into small pieces and rinsed with cold PBS to remove residual blood. Liver tissue was homogenized in 2% SDS and 100 mM Tris-HCl lysing buffer (pH = 7.4), with protease inhibitor, by blade homogenizer. The homogenate was sonicated to shear DNA, and the lysate was centrifuged at 20,000G for 15 min to remove any insoluble content. A 10 fold volume of cold acetone was added to the supernatant, and the protein was precipitated at −20^o^C overnight. Protein precipitant was collected by centrifugation at 20,000xg for 15 min, and rinsed with cold ethanol/acetone (50%:50%, v/v) twice to remove trace SDS. Dried protein precipitant was dissolved with 8M urea in PBS, and protein concentration was measured by DC Protein Assay (Bio-Rad). Liver lysates from mice in the same group were pooled, and 500 μg of protein from pooled lysate was used as the starting material for the proteomics study. Protein was denatured, reduced by adding 10 mM DTT, and incubated at 56^o^C for 45 min. Reduced protein was alkylated by adding 20 mM IAA and incubated at room temperature in the dark for 30 min. The sample solution was diluted 5 times with 100 mM TEAB, and trypsin was added at 1:50 ratio. The protein was digested by incubation at 37 °C overnight. After digestion, peptides from control and GlcNAc-treated liver samples were labelled with light and heavy stable-isotope dimethyl respectively, as described elsewhere^58^. Labelled peptides were mixed and desalted with a C18 cartridge. 10% of the elution was dried by Speed Vac for total proteome analysis, and the rest was used for glycopeptide enrichment by hydrophilic interaction chromatography (HILIC SPE). The elutions from HILIC SPE were divided into two aliquots: one aliquot was dried by Speed Vac, and the other was incubated with peptide-N-glycosidase F (PNGase F) for deglycosylation. Both deglycosylated peptides and intact glycopeptides were analyzed by LC-MS/MS with an Orbitrap-Elite mass spectrometer. Quantification of total proteome and N-glycosylated sites were achieved with Maxquant, and results were processed with Perseus^59^. Intact glycopeptides were identified and quantified by matching the Y1 ion from the MS/MS spectrum to the deglycosylated peptides identified with Mascot, and extracting the peak of the peptides precursor^34^. Glycan compositions of intact glycopeptides were validated manually after software analysis.

### Cell Culture

AML12 immortal hepatocytes were purchased from ATCC. 3T3-L1 fibroblast cells were kindly provided by Dr. Amira Klip (Sick Kids Research Institute). Mouse primary hepatocytes were isolated as previously described^13^. Intracellular lipid accumulation in lipid droplets was detected using the lipophilic fluorescent probe BODIPY 493/503. For quantitative microscopic fluorescence imaging, cells were seeded in a 96-well plate in regular media at 37 °C and 5% CO_2_, and treated with GlcNAc for indicated times. Cells were fixed for 15 min with 4% paraformaldehyde, washed with PBS, and incubated for 1 h at room temperature in 50 μl PBS containing Hoechst 33342, and either BODIPY 493/503, or Alexa Fluor-488-conjugated ConA or L-PHA. Staining per cell were quantified using an IN Cell Analyzer 1000 automated fluorescence imaging system. 2-NBD-Glc uptake was measured by mean fluorescence intensity (MFI) using Beckman Coulter Gallios flow cytometer and analyzed using Kaluza analysis software.

### Statistical Analysis

All data are expressed as mean ± standard error of the mean. Statistical significance was determined using Microsoft’s Excel or GraphPad Prism software. In all experiments a *p*-value of 0.05 or less was considered to be statistically significant. Metaboanalyst (http://www.metaboanalyst.ca/MetaboAnalyst/), a comprehensive online software suite for metabolomic data analysis was used to generate the Principle Component Analysis (PCA) scatter plot^60^.

## Additional Information

**Data availability:** The LC-MS/MS metabolomics data has been deposited at http://www.peptideatlas.org/PASS/PASS00836

**How to cite this article**: Ryczko, M. C. *et al.* Metabolic Reprogramming by Hexosamine Biosynthetic and Golgi N-Glycan Branching Pathways. *Sci. Rep.* **6**, 23043; doi: 10.1038/srep23043 (2016).

## Supplementary Material

Supplementary Information

## Figures and Tables

**Table 1:** Serum biochemistry in GlcNAc-treated and untreated mice on 9% fat diet.

	fasted 18 h		fed *ad libitum*	
	control	GlcNAc	control	GlcNAc
**ALT (IU/L)**	102.8 ± 38.98	99.20 ± 27.51	24.75 ± 2.213	41.75 ± 10.40
**Triglycerides (mmol/L)**	1.222 ± 0.2203	1.268 ± 0.1545	0.9860 ± 0.09563	1.046 ± 0.07019
**Cholesterol (mmol/L)**	2.250 ± 0.5781	3.640 ± 0.2272*	3.500 ± 0.4528	4.100 ± 0.1483
**Chloride (mmol/L)**	100.4 ± 0.5099	98.80 ± 0.6633	100.6 ± 0.6782	99.00 ± 0.8367
**Sodium/Potassium Ratio**	20.40 ± 0.8124	21.40 ± 0.9798	21.60 ± 0.8718	20.60 ± 1.470
**Glucose (mM)**	4.225 ± 0.1493	6.400 ± 0.3342**	7.840 ± 0.3558	7.700 ± 0.4743
**Insulin (pM)**	39.17 ± 7.522	314.7 ± 84.37*	334.7 ± 57.20	409.1 ± 97.21
**Glucagon (pM)**	33.65 ± 2.348	22.45 ± 2.548*	18.75 ± 4.543	22.63 ± 4.146
**Insulin/Glucagon Ratio**	2.590 ± 1.073	14.59 ± 4.485*	30.42 ± 4.240	14.90 ± 0.9945*
**HOMA2-IR**	1.600 ± 0.8820	5.425 ± 1.276*	N/A	N/A
**Leptin (pM)**	313.1 ± 102.4	1040 ± 94.97**	900.5 ± 96.63	1412 ± 75.67*
**Adiponectin (ng/ml)**	23.35 ± 0.4644	23.50 ± 0.3549	25.62 ± 0.3820	24.12 ± 0.3151*

Serum biochemistry was analyzed after 30 weeks with or without 0.5 mg/ml oral GlcNAc supplementation in the drinking water, at which time the mice were 36 weeks of age. Data shown are mean ± SEM, n = 4–5, with statistical significance indicated as *p < 0.05 and **p < 0.01 (2-tailed, unpaired Student’s *t*-test) for GlcNAc-treated versus untreated control group in either 18 h fasted or fed *ad libitum* mice on 9% fat diet. ALT alanine aminotransferase, HOMA-IR homeostatic model assessment-insulin resistance.

**Table 2:** Global analysis of GlcNAc content in liver N-glycans.

Diet	GlcNAc content (GlcNAc-treated/control)#	Unique glycopeptides^	*p*-value
**4% fat**	1.54	361	<0.0001
**4% fat, fasted**	1.99	394	<0.0001
**9% fat**	1.52	292	<0.0001
**9% fat, fasted**	1.54	218	0.2785

^Sequence-identified N-glycan bearing glycopeptides were compared from livers of control and GlcNAc-treated mice. Hepatic glycopeptide pool from control and GlcNAc-treated mice was differentially labelled with light (control) and heavy (GlcNAc-treated) stable-isotope dimethyl^58^, followed by LC-MS/MS analysis using intact and deglycosylated glycopeptides^34^. ^#^The GlcNAc content of N-glycans from hepatic glycoproteins of control and GlcNAc-treated mice on each glycopeptide were compared as a ratio (null hypothesis is 1). Sign test with probability of 0.5 and two-tail *p*-value was performed.
