# Supplementary material for: Metabolic Reprogramming by Hexosamine Biosynthetic and Golgi N-Glycan Branching Pathways
Source: Sci Rep. 2016 Mar 14;6:23043. doi: 10.1038/srep23043 (PMC4789752; doi:10.1038/srep23043)
Supplement: Supplementary Information [file srep23043-s1.pdf]

Supplementary Information

Metabolic Reprogramming by Hexosamine Biosynthetic and Golgi N-Glycan Branching Pathways

Michael C. Ryczko[1,2], Judy Pawling[1], Rui Chen[3,4], Anas M. Abdel Rahman[1,5], Kevin Yau[1], Julia K. Copeland[6], Cunjie Zhang[1], Anu Surendra[6], David S. Guttman[6], Daniel Figeys[3,7], and James W. Dennis[1,2,8,*]

[1]Lunenfeld-Tanenbaum Research Institute, Mount Sinai Hospital, 600 University Ave., Toronto ON M5G 1X5, Canada

[2]Department of Molecular Genetics, University of Toronto, Toronto ON M5S 1A8, Canada

[3]Ottawa Institute of Systems Biology, Department of Biochemistry, Microbiology and Immunology, Faculty of Medicine, University of Ottawa, Ottawa ON K1H 8M5, Canada

[4]CAS Key Lab of Separation Sciences for Analytical Chemistry, National Chromatographic Research and Analysis Center, Dalian Institute of Chemical Physics, Chinese Academy of Sciences, Dalian 116023, China

[5]Department of Genetics, Research Center, King Faisal Specialist Hospital and Research Center, Riyadh 11211, Kingdom of Saudi Arabia

[6]Centre for the Analysis of Genome Evolution & Function, University of Toronto, Toronto ON M5S 3B2, Canada

[7]Department of Chemistry, Faculty of Science, University of Ottawa, Ottawa ON K1N 6N5, Canada

[8]Department of Laboratory Medicine and Pathobiology, University of Toronto, Toronto ON M5S 1A8, Canada

Supplementary Methods

16S rRNA Gene Sequencing and Analysis of Bacterial Gut Microbiome.

Age and weight matched young male mice were supplemented with 0.5 mg/ml GlcNAc in drinking water and placed on 9% fat diet for 2 weeks. Before GlcNAc supplementation, and 2 weeks after, individual mice were transferred to a fresh disinfected cage and fecal pellets (~200 mg) collected and immediately frozen on dry ice for further analysis. Power Soil DNA Isolation kit from MO BIO Laboratories Inc. was used to extract microbial genomic DNA from fecal samples for each individual mouse. The V4 hypervariable region of the 16S rRNA gene was amplified using a universal forward sequencing primer and a uniquely barcoded reverse sequencing primer to allow for multiplexing[1]. Primers contained an adapter sequence to bind the amplicons to the Illumina flow cell. PCR-based library construction was performed in triplicate 25 µl solutions containing 1X KAPA2G Robust HotStart ReadyMix, 600 nM each of primer, and 1 µl of DNA template. For every PCR reaction sterile dH2O was used as a negative control to ensure no contaminating DNA was present. PCR conditions were 95°C for 3 min, followed by 17 cycles of 95°C for 15 s, 50°C for 15 s, 72°C for 15 s and were completed at 72°C for 5 min. All PCR reactions were run on a 1% agarose TBE gel to visualize the amplification and approximate DNA quantity. Triplicates were pooled together and then combined by approximately even concentrations based on the gel images to create the final library. The final library was purified using 0.8X volumes of Agencourt AMPure XP beads (Beckman Coulter, Indianapolis, IN) according to the manufacturer's protocol and quantified using the Qubit Fluorometer. The final library was prepared according to the MiSeq user guide, diluted to a concentration of 7 pM and combined with a 15% PhiX control. Sequencing was performed using the V2 (150bp x 2) chemistry and sequenced on the Illumina MiSeq (Illumina, San Diego, CA).

The UPARSE pipeline, available through USERACH, was used for sequence analysis[2,3]. Sequences were assembled and quality trimmed using –fastq_mergepairs and –fastq_filter, with a –fastq_maxee set at 1.0 and 0.5, respectively. Following the UPARSE pipeline, merged pairs were then de-replicated and sorted to remove singletons. Sequences were clustered into operational taxonomic units (OTUs) at 97% identity. Chimeras were detected and removed using the –uchime_ref reference based method and the Ribosomal Database Project (RDP) 16S database, derived from the RDP training set version 9, accessed through USEARCH[4].

Assembled sequences were then mapped back to the chimera-free OTUs. Taxonomy assignment was executed using -utax available through USEARCH. A minimum confidence of 0.5 standard deviations away from the mean P-value was used at each taxonomic level. OTU sequences were aligned using MUSCLE, accessed through QIIME[5]. A phylogenetic tree of the aligned sequence data was made using FastTree[6].

The mapped sequences and the taxonomic information were converted to a tab-delimited format using the python scripts available through USEARCH. This file was converted into a BIOM format file using QIIME. Low abundance OTUs (<0.005% RA) were removed from the analysis[7]. The data was rarefied to 70,000 sequences per sample, as determined by rarefaction curves and the minimum sequence coverage. Relative abundances of the community members were determined using the rarefied data and summarized at each taxonomic level. Alpha and beta diversity were calculated using the rarefied OUT-level data in QIIME. Statistical analyses were performed using SPSS Statistics version 20.0.0 (IBM, United States). The relative abundances of specific taxa were analyzed between treatment groups and before and after treatments. The variance of means was assessed for the relative abundances of each taxa investigated within each category of analysis. A student's t-test was used to compare the relative abundances of these taxa between treated and untreated samples and a paired t-test was used to compare the relative abundances of these taxa before and after treatment.

Supplementary References

1 Caporaso, J. G. *et al.* Ultra-high-throughput microbial community analysis on the Illumina HiSeq and MiSeq platforms. *ISME J* **6**, 1621-1624 (2012).

2 Edgar, R. C. Search and clustering orders of magnitude faster than BLAST. *Bioinformatics* **26**, 2460-2461 (2010).

3 Edgar, R. C. UPARSE: highly accurate OTU sequences from microbial amplicon reads. *Nat Methods* **10**, 996-998 (2013).

4 Wang, Q., Garrity, G. M., Tiedje, J. M. & Cole, J. R. Naive Bayesian classifier for rapid assignment of rRNA sequences into the new bacterial taxonomy. *Appl Environ Microbiol* **73**, 5261-5267 (2007).

5 Caporaso, J. G. *et al.* QIIME allows analysis of high-throughput community sequencing data. *Nat Methods* **7**, 335-336 (2010).

6 Price, M. N., Dehal, P. S. & Arkin, A. P. FastTree: computing large minimum evolution trees with profiles instead of a distance matrix. *Mol Biol Evol* **26**, 1641-1650 (2009).

7 Bokulich, N. A. *et al.* Quality-filtering vastly improves diversity estimates from Illumina amplicon sequencing. *Nat Methods* **10**, 57-59 (2013).

Figure S1

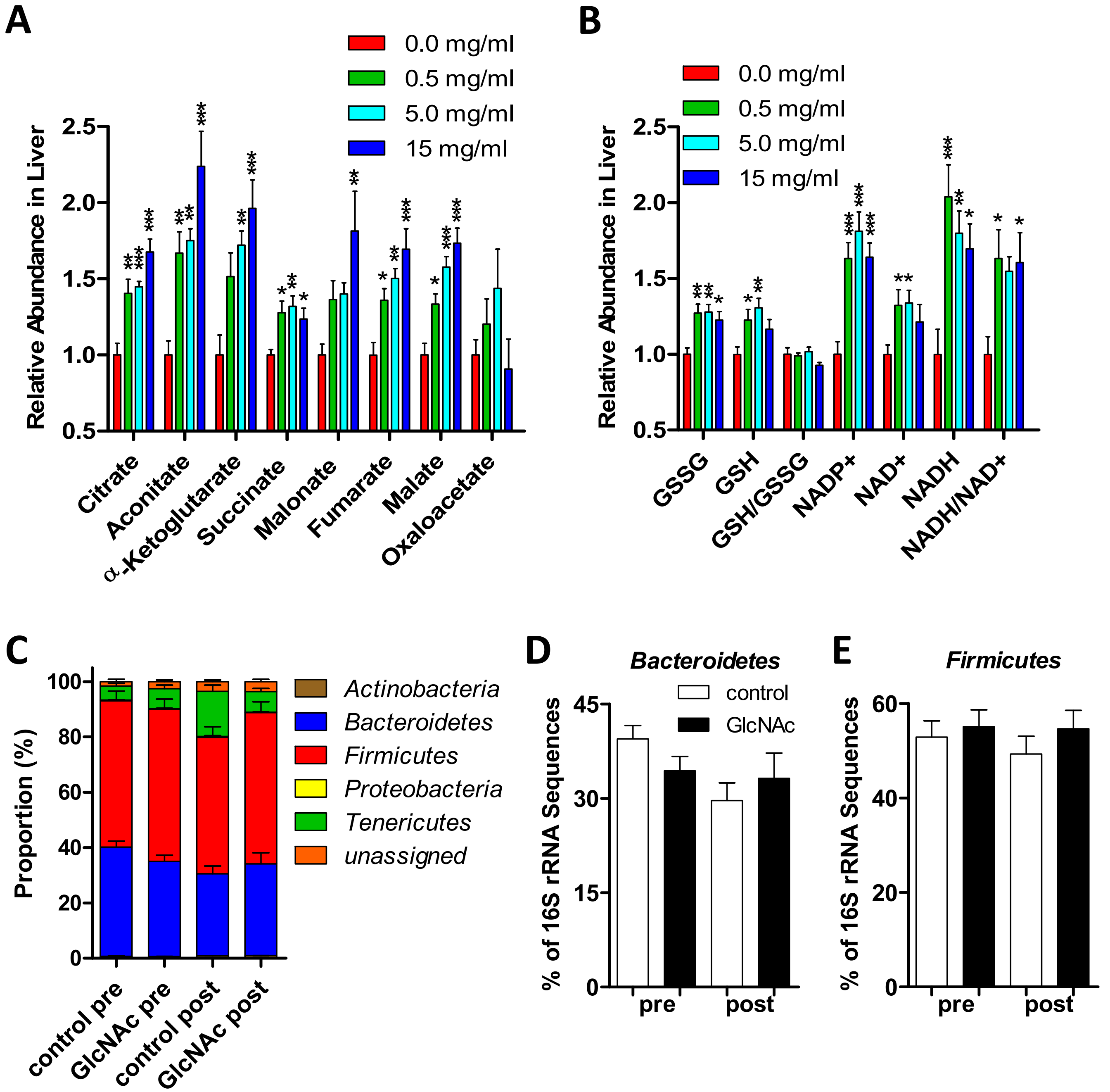

Figure S1: Hepatic metabolites and gut microbiome in GlcNAc supplemented mice. Relative abundance of liver metabolites in mice on 4% fat diet and GlcNAc supplemented in drinking water at 0, 0.5, 5.0 and 15 mg/ml. Steady-state liver metabolites in **(A)** tricarboxylic acid (TCA) cycle, and **(B)** oxidized and reduced forms and ratios of glutathione and nicotinamide adenine dinucleotides. Data shown are mean ± SEM, n=10, with significant differences represented vertically as **p*<0.05, ***p*<0.01 and ****p*<0.001. **(C)** Relative abundances of phylum-level taxa in the gut microbiome of control and GlcNAc pre treated and post treated mice, n= 5-10. Relative abundance of **(D)** *Bacteroidetes* and **(E)** *Firmicutes*. 16S rRNA sequencing analysis was performed on feces of age and weight matched young male mice treated with 0.5 mg/ml GlcNAc in drinking water and maintained on 9% fat diet for 2 weeks. Data shown are mean ± SEM, n= 5-10.

Figure S2

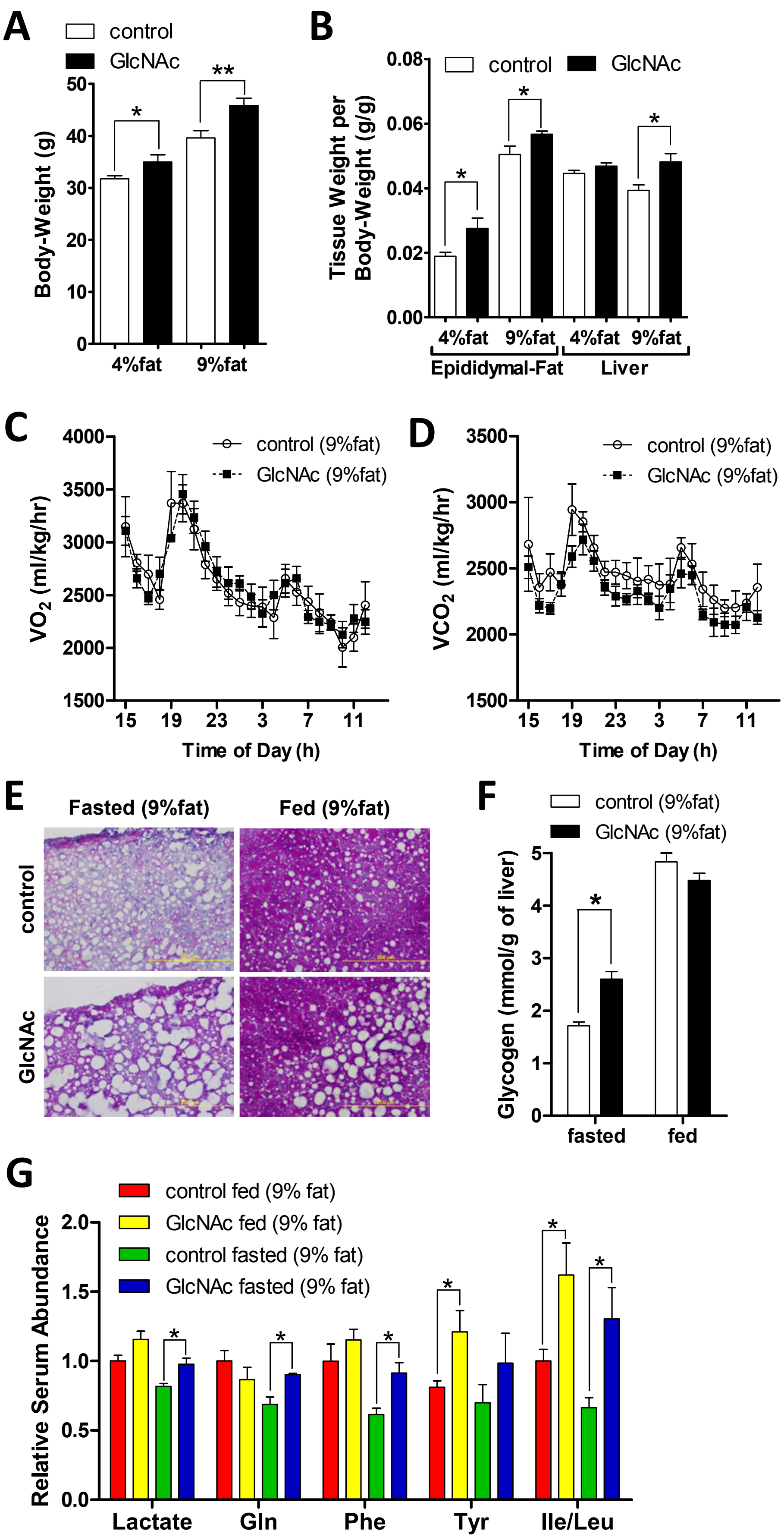

A
control
GlcNAc
Body-Weight (g)
4%fat
9%fat
B
control
GlcNAc
Tissue Weight per Body-Weight (g/g)
4%fat
9%fat
Epididymal-Fat
Liver
C
control (9%fat)
GlcNAc (9%fat)
VO2 (ml/kg/hr)
Time of Day (h)
D
control (9%fat)
GlcNAc (9%fat)
VCO2 (ml/kg/hr)
Time of Day (h)
E
Fasted (9%fat)
Fed (9%fat)
control
GlcNAc
F
control (9%fat)
GlcNAc (9%fat)
Glycogen (mmol/g of liver)
fasted
fed
G
control fed (9% fat)
GlcNAc fed (9% fat)
control fasted (9% fat)
GlcNAc fasted (9% fat)
Relative Serum Abundance
Lactate
Gln
Phe
Tyr
Ile/Leu

Figure S2. Oral GlcNAc promotes weight-gain. (A) Body-weight and **(B)** epididymal-fat and liver weight normalized to body-weight for wild-type C57BL/6 male mice after 30 weeks on 4% or 9% fat diet with and without GlcNAc supplemented drinking water at 0.5 mg/ml. Data shown are mean ± SEM, n=10, *$p<0.05$ and **$p<0.01$ GlcNAc treated versus control on either 4% or 9% fat diet. **(C)** Oxygen consumption rate and **(D)** carbon dioxide emission rate in mice maintained on 9% diet with and without GlcNAc supplementation. Data shown are mean ± SEM, n=5. **(E)** Representative images of liver histology sections stained with periodic acid-Schiff, with glycogen deposits detected as purple-magenta areas and white areas as lipid droplets, obtained from mice on 9% fat diet, fed *ad libitum* or fasted for 18 hours with and without 0.5 mg/ml GlcNAc in drinking water for 30 weeks. **(F)** Quantification of liver glycogen content. Data shown are mean ± SEM, n=5, *$p<0.05$ versus control. **(G)** Steady-state relative abundance of metabolites in blood serum from mice maintained on 9% fat diet and oral GlcNAc for 30 weeks. Data shown are mean ± SEM, n=4-5, analyzed with 2-tailed unpaired Student's t-test, *$p<0.05$ GlcNAc treated versus control in either fed *ad libitum* or fasted conditions.

Figure S3

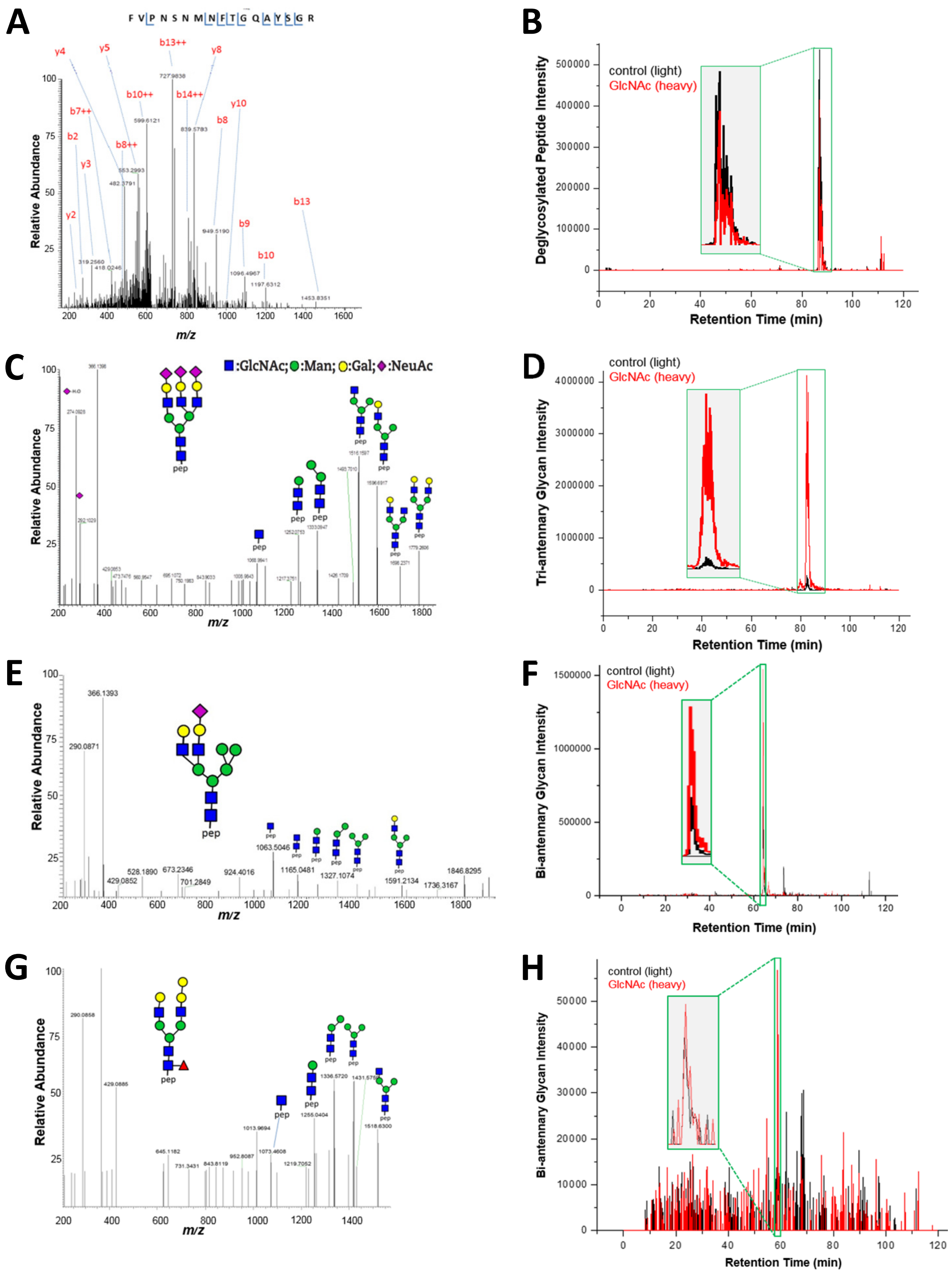
A
FVPNSNMNFTGQAYSGR
Relative Abundance
m/z
B
control (light)
GlcNAc (heavy)
Deglycosylated Peptide Intensity
Retention Time (min)
C
:GlcNAc; :Man; :Gal; :NeuAc
Relative Abundance
m/z
D
control (light)
GlcNAc (heavy)
Tri-antennary Glycan Intensity
Retention Time (min)
E
Relative Abundance
m/z
F
control (light)
GlcNAc (heavy)
Bi-antennary Glycan Intensity
Retention Time (min)
G
Relative Abundance
m/z
H
control (light)
GlcNAc (heavy)
Bi-antennary Glycan Intensity
Retention Time (min)

Figure S3. Oral GlcNAc increases tri-antennary N-glycan branching on glycosite Asn89 of CEACAM1 hepatic transmembrane glycoprotein. (A) Matching the Y1 ion (peptide + GlcNAc) from MS/MS spectrum to the list of deglycosylated peptides identified by Mascot database search with accurate molecular weight and retention time, identified the peptide sequence as Asn89 of CEACAM1 transmembrane glycoprotein. **(B)** The extracted ion chromatogram (XIC) peak area for control (light) and GlcNAc (heavy) labelled precursor from deglycosylated CEACAM1 peptides. With a GlcNAc treated to control ratio of 1.68, no significant difference was found at the peptide level. **(C)** Annotated MS/MS spectrum of heavy-labelled intact glycopeptide identified with tri-antennary N-glycan structure. Terminal sialylation could be verified by the existence of oxonium ion with m/z 292 and 274. **(D)** XIC for control and GlcNAc labelled peptide precursor from full MS scan indicates the abundance of tri-antennary glycopeptide is higher in liver lysates from GlcNAc treated mice, with a GlcNAc to control ratio of 21. **(E)** Hybrid bi-antennary N-glycan structure with unsubstituted terminal mannose residues. **(F)** XIC from control and GlcNAc bi-antennary hybrid N-glycan with mannose, with a peak area ratio of 1.21. **(G)** Complex bi-antennary N-glycan structure. **(H)** XIC for control and GlcNAc bi-antennary N-glycan with a GlcNAc to control ratio of 1.17. Symbols and colors for N-glycans used according to nomenclature from Consortium for Functional Glycomics.

Figure S4

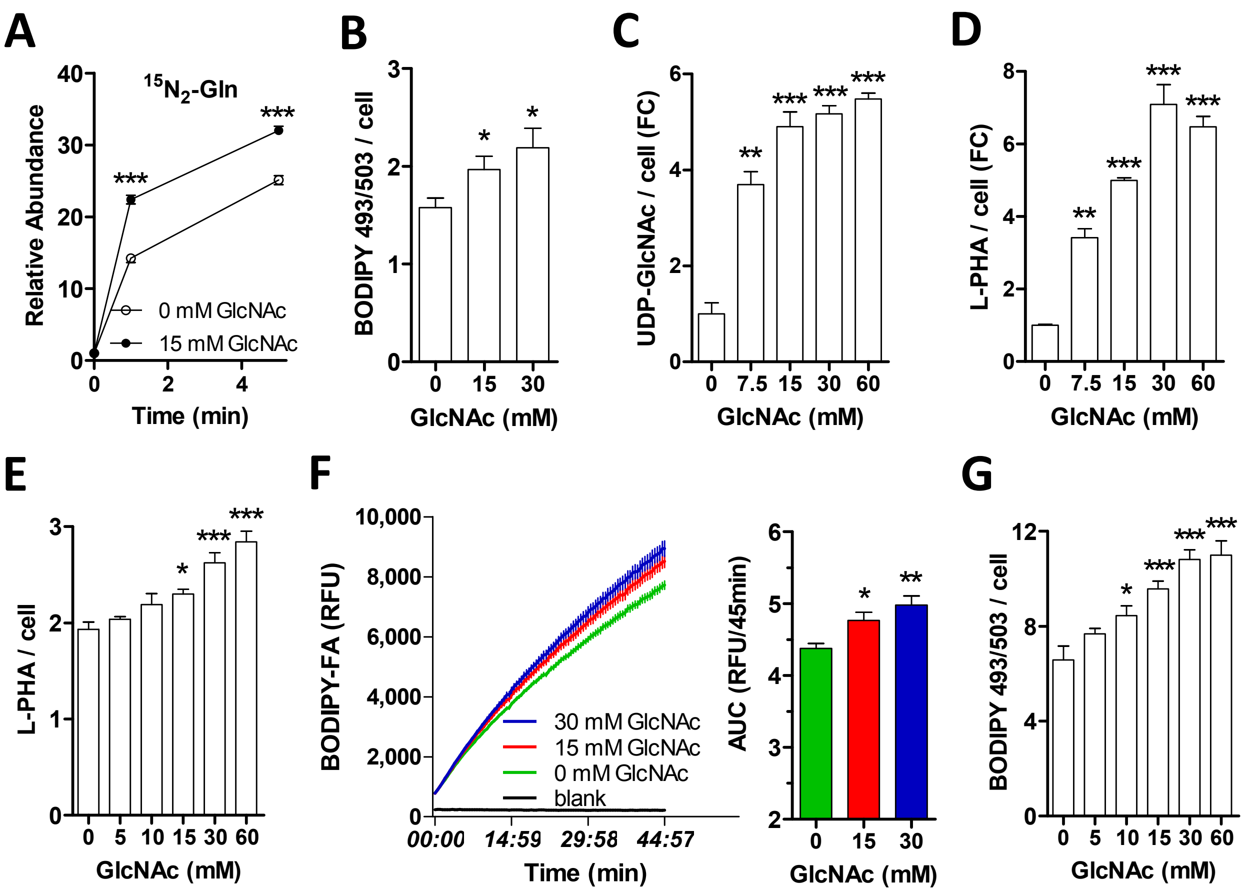

Figure S4. GlcNAc increases nutrient uptake and lipid accumulation. (A) Glutamine uptake in HeLa cells treated with GlcNAc for 20 h, pulsed with $^{15}N_2$-Gln, and quantified using targeted LC-MS/MS. **(B)** Lipid droplet content in HeLa cells treated with GlcNAc. Fold change (FC) in **(C)** UDP-GlcNAc intracellular pool and **(D)** cell surface lectin L-PHA binding as a function of GlcNAc treatment in HeLa cells. Data shown are mean ± SEM, analyzed with 2-tailed unpaired Student's t-test with significant differences represented as *$p<0.05$, **$p<0.01$ and ***$p<0.01$ versus untreated control. **(E)** Mgat5-dependent cell surface N-glycan branching detected with L-PHA in 3T3-L1 adipocytes. **(F)** Kinetic reading monitoring uptake of exogenous non-esterified long-chain fatty-acid analog (BODIPY-FA) quantified as area under the curve (AUC) for Relative Fluorescence Units (RFU) in 3T3-L1 adipocytes. **(G)** Intracellular lipid droplet content in 3T3-L1 adipocytes treated with GlcNAc. Data are mean ± SEM, analyzed by *t*-test or one-way ANOVA followed by Dunnett's test, significant differences represented as *$p<0.05$, **$p<0.01$ and ***$p<0.001$.

Figure S5

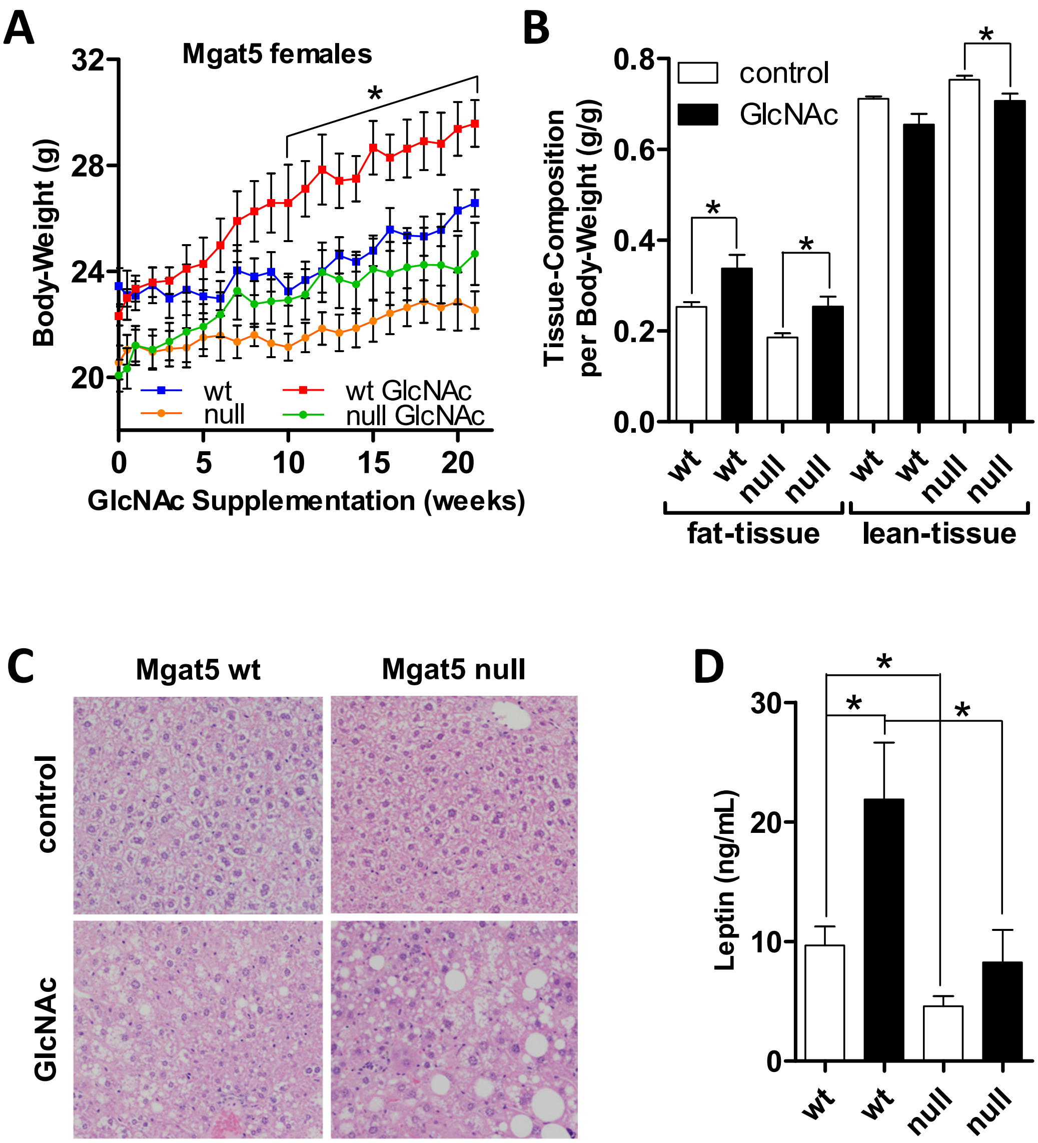

Figure S5. Oral GlcNAc promotes lipid storage in female Mgat5$^{+/+}$ and Mgat5$^{-/-}$ mice. (A) Change in body-weight in mice on 9% fat diet supplemented with 0.5 mg/ml oral GlcNAc in drinking water for 21 weeks. Data shown are mean ± SEM, with statistical significance indicated as *p<0.05 for GlcNAc versus control Mgat5$^{+/+}$ mice, n=4-6. **(B)** Fat-tissue and lean-tissue mass to body-weight ratio, measured by EchoMRI. Data shown represent mean ± SEM, n=4-6, with statistical significance indicated as *p<0.05 versus untreated control for the same genotype. **(C)** Representative images of liver histology sections stained with haematoxylin and eosin, with white round areas showing lipid accumulation, obtained from mice on 9% fat diet with 0.5 mg/ml GlcNAc in drinking water for 23 weeks. **(D)** Serum concentration of leptin in Mgat5$^{+/+}$ and Mgat5$^{-/-}$ mice supplemented with GlcNAc. Data shown are mean ± SEM, n=4-6, with statistical significance indicated as *p<0.05.
